# mTOR is critical for intestinal T-cell homeostasis and resistance to *Citrobacter rodentium*

**DOI:** 10.1038/srep34939

**Published:** 2016-10-12

**Authors:** Xingguang Lin, Jialong Yang, Jinli Wang, Hongxiang Huang, Hong-Xia Wang, Pengcheng Chen, Shang Wang, Yun Pan, Yu-Rong Qiu, Gregory A. Taylor, Bruce A. Vallance, Jimin Gao, Xiao-Ping Zhong

**Affiliations:** 1 Department of Pediatrics, Division of Allergy and Immunology, Duke University Medical Center, Durham, NC 27710, USA; 2 School of Laboratory Medicine, Wenzhou Medical University, Wenzhou, Zhejiang 325035, China; 3 Department of Oncology, Nanfang Hospital, Southern Medical University, Guangzhou, Guangdong 510515, China; 4 Laboratory Medicine Center, Nanfang Hospital, Southern Medical University, Guangzhou, Guangdong 510515, China; 5 Geriatric Research, Education, and Clinical Center, VA Medical Center, Durham, NC 27705, USA; 6 Department of Medicine, Division of Geriatrics, and Center for the Study of Aging and Human Development, Duke University Medical Center, Durham NC 27710, USA; 7 Department of Molecular Genetics and Microbiology Duke University Medical Center, Durham NC 27710, USA; 8 Division of Gastroenterology, Department of Pediatrics, Child and Family Research Institute and the University of British Columbia, Vancouver, British Columbia V6H 3V4, Canada; 9 Department of Immunology, Medical Center, Durham, NC 27710, USA; 10 Hematologic Malignancies and Cellular Therapies Program, Duke Cancer Institute, Duke University Medical Center, Durham, NC 27710, USA

## Abstract

T-cells play an important role in promoting mucosal immunity against pathogens, but the mechanistic basis for their homeostasis in the intestine is still poorly understood. We report here that T-cell-specific deletion of mTOR results in dramatically decreased CD4 and CD8 T-cell numbers in the lamina propria of both small and large intestines under both steady-state and inflammatory conditions. These defects result in defective host resistance against a murine enteropathogen, *Citrobacter rodentium*, leading to the death of the animals. We further demonstrated that mTOR deficiency reduces the generation of gut-homing effector T-cells in both mesenteric lymph nodes and Peyer’s patches without obviously affecting expression of gut-homing molecules on those effector T-cells. Using mice with T-cell-specific ablation of Raptor/mTORC1 or Rictor/mTORC2, we revealed that both mTORC1 and, to a lesser extent, mTORC2 contribute to both CD4 and CD8 T-cell accumulation in the gastrointestinal (GI) tract. Additionally, mTORC1 but not mTORC2 plays an important role regulating the proliferative renewal of both CD4 and CD8 T-cells in the intestines. Our data thus reveal that mTOR is crucial for T-cell accumulation in the GI tract and for establishing local adaptive immunity against pathogens.

The gastrointestinal (GI) tract encounters an array of microbes including members of normal microflora as well as enteric pathogens. T-cells are a major population of immune cells found within the intestinal mucosa that play important functions in intestinal tissue homeostasis as well as a defense against invading pathogens. CD4 T-cells play a critical role in host defense against enteropathogens such as *Citrobacter rodentium*, an attaching/effacing bacterium and a natural pathogen of mice^1^,^2^,^3^,^4^. Intestinal T-cells also contribute to inflammatory bowel diseases, such as Crohn’s disease and ulcerative colitis, as well as intestinal food intolerances, such as celiac disease. The intestinal lamina propria (LP) harbors large numbers of αβ T-cells. Homing T-cells to the intestine, as well as the maintenance of these cells within the intestines, are complicated processes that involve the initial activation of naïve T-cells in the mesenteric lymph nodes (mLNs) and Peyer’s patches (PPs). This activation imprints these cells with properties necessary for gut tropism^5^,^6^,^7^,^8^, including upregulation of chemokine receptors such as CCR9^9^, the orphan G-protein-coupled receptor 15 (GPR15)^10^,^11^, integrin α_4_β_7_^12^,^13^, and CD103^14^,^15^. These imprinted cells re-enter the blood circulation through the thoracic duct before homing to the intestines. Interaction of CCR9 with its ligand CCL25 expressed by small intestinal (SI) epithelial cells increases affinity of integrin α_4_β_7_ to its ligand for mucosal vascular addressin cell adhesion molecule-1 (MadCAM-1) expressed on intestinal LP vascular endothelium. Such interaction leads to the migration of effector T-cells to the SI-LP compartment^16^,^17^. In contrast, T-cell homing to the large intestinal (LI) LP compartment does not require CCR9 but is instead mediated by GPR15 and integrin α_4_β_7_^10^,^10^. In both the SI-LP and LI-LP compartments, T-cells use signals from a variety of cell types including dendritic cells, macrophages, stromal cells, and epithelial cells, as well as the extracellular matrix and pathogen-associated molecular patterns and metabolites such as short-chain fatty acids from the microbiota to maintain their survival and proliferative renewal. Intestinal T-cells also detect pathogen-associated molecular patterns and metabolites such as short-chain fatty acids from the microbiota for similar purposes^18^,^19^,^20^. However, little is known about the specific signaling events that promote T-cell accumulation/homeostasis in the gut.

The serine/threonine kinase mTOR plays important roles in cell growth, proliferation, autophagy, survival, and metabolism. It forms at least two signal complexes, the Raptor containing mTORC1 and Rictor containing mTORC2, which perform distinct functions. mTORC1 phosphorylates multiple downstream molecules such as S6K1 and 4EBP-1 to promote protein, nuclei acid, and lipid synthesis. mTORC2 phosphorylates Akt, PKCα and PKCθ, and SGK1 to regulate cell survival, nutrient uptake, and actin polymerization^21^. mTOR can be activated in T-cells following the engagement of multiple receptors such as the T-cell receptor and cytokine receptors via multiple downstream pathways^22^,^23^,^24^. Evidence has demonstrated that mTOR and its tight regulation are central for thymopoiesis^25^,^26^, T-cell homeostasis^27^,^28^,^29^,^30^, T-cell activation and effector/memory differentiation^31^,^32^,^33^,^34^,^35^,^36^, and regulatory T-cell function^37^,^38^, as well as for iNKT-cell generation, effector lineage fate decision, and anti-tumor immunity^39^,^40^,^41^,^42^,^43^,^44^,^45^. Although a recent report has revealed that mTORC1 is important for the generation of the mucosal CD8 effector and resident memory T-cells following viral infection^46^, the potential role of mTORC1 and mTORC2 in regulating CD4 and CD8 T-cell accumulation/homeostasis in the intestines under steady state and during an enteric bacterial infection remains unknown.

We report here that mTOR plays crucial roles for both CD4 and CD8 T-cell accumulation/homeostasis in the intestines. The T-cell specific deletion of mTOR led to a severe decrease in T-cell numbers in both the SI-LP and LI-LP compartments. Consistent with the importance of T-cells in host defense against enteropathogens, mTOR deficiency leads to impaired host resistance to *C. rodentium* infection. Moreover, both mTORC1 and mTORC2 contribute to T-cell accumulation/homeostasis in the gut, while mTORC1 but not mTORC2 proved critical for the proliferative renewal of SI-LP and LI-LP T-cells.

## Results

### mTOR deficiency in T-cells impaired host resistance to *Citrobacter rodentium*

**Figure 1:**
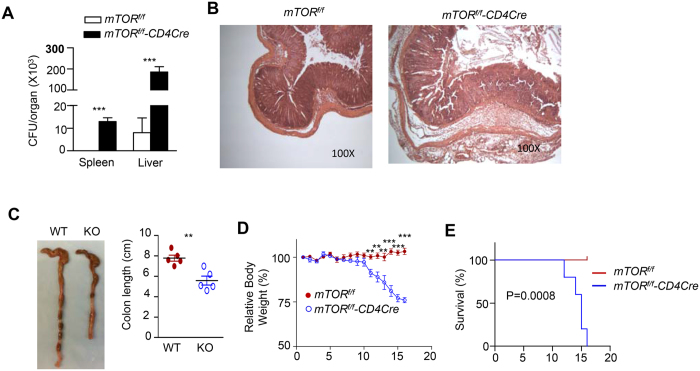
Susceptibility to *C. rodentium* in mTORKO mice. *mTOR*^*f/f*^*-CD4Cre* mice and *mTOR*^*f/f*^ (WT) control mice were intragatrically injected with 2.5 × 10^8^ CFU *C. rodentium* in 100 μl PBS. Mice were weighted daily after infection. Mice with weight loss greater than 25% were considered moribund and were euthanized; otherwise, mice were euthanized on day16. Displayed are: (**A**) *C. rodentium* titers in the spleen and liver. Bar graphs represent mean ± SEM of colony forming unit (CFU; Ctrl, n = 6; KO, n = 5). ***p < 0.001 determined by two-tailed Student’s *t*-test. (**B**) Representative H&E stained tissues. (**C**) Representative photographs of colons, and average colon lengths. Error bars represent SEM; **p < 0.001 as determined by two-tailed unpaired Student’s *t*-test. (**D**) Body weights after infection. **p < 0.01, ***p < 0.001 determined by two-tailed Student’s *t*-test. (**E)** Kaplan-Meyer survival plot. Data shown are representative of two experiments. P value was determined by Log-rank (Mantel-Cox) test.

To investigate whether mTOR in T-cells plays an important role in mucosal immunity against microbial pathogens, we infected T-cell-specific mTOR deficient, *mTOR*^*f/f*^*-CD4Cre,* (mTORKO) mice and *mTOR*^*f/f*^ (WT) control mice with *C. rodentium* via intragastric inoculation. *C. rodentium* is an extracellular, gram-negative bacterium that instigates a self-resolving inflammatory response in immunocompetent mice. The clearance of *C. rodentium* requires strong CD4 cell-mediated Th1 and Th17 responses^2^,^47^. While control mice were capable of eradicating the pathogen and recovering from the infection, mTORKO mice failed to control the infection, as reflected by high bacterial burdens in the spleen and liver (Fig. 1A), severe colonic inflammation (Fig. 1B), increased colonic shortening (Fig. 1C), progressive weight loss (Fig. 1D), and eventual death (Fig. 1E). Thus, deficiency of mTOR in T-cells resulted in defective mucosal immunity against the bacterial pathogen.

### Severe decreases in LP T-cell populations *mTOR* ^*f/f*^-*CD4Cre* mice

**Figure 2:**
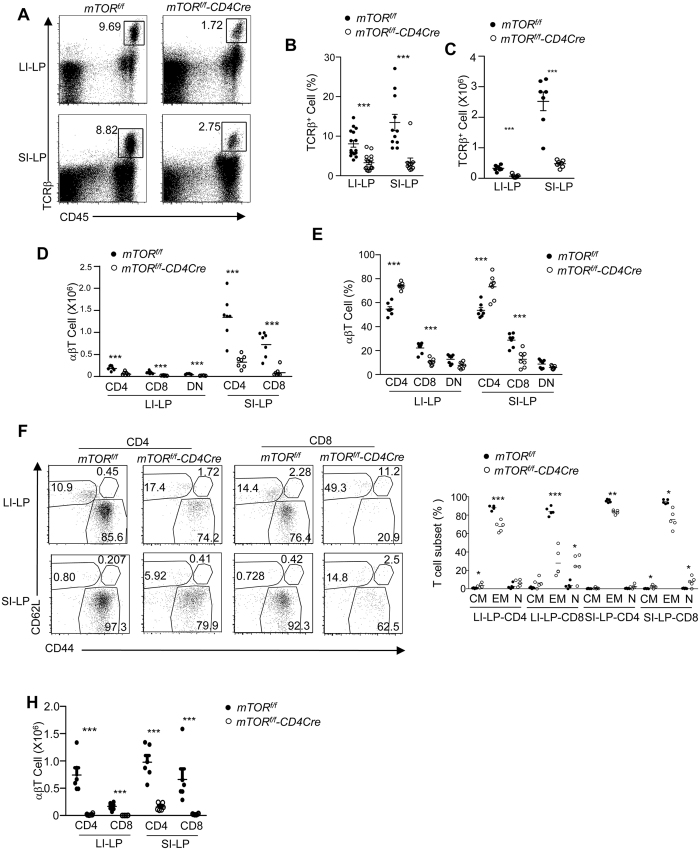
Decreases in intestinal LP T-cells in *mTOR*^*f/f*^*-CD4Cre* mice. Single SI-LP and LI-LP cell preparations from *mTOR*^*f/f*^ and *mTOR*^*f/f*^-*CD4Cre* mice were stained with the indicated antibodies and analyzed by flow cytometry. Shown are: (**A**) Representative dot-plots of CD45 and TCRβ staining. (**B**,**C**) Scatter graphs showing the mean ± SEM of αβT-cell percentages (**B**) and numbers (**C**,**D**) CD4. CD8, and CD4^−^CD8^−^ (DN) T-cell numbers. (**E**) Percentages of CD4, CD8, and DN subsets within αβT-cells. (**F**) Dot-plots showing CD44 and CD62L staining in gated CD4 and CD8 T-cells. (**G**) Percentages (mean ± SEM) of T_N_, T_EM_, and T_CM_ cells within CD4 and CD8 T-cells. (**H**) LI-LP and SI-LP T-cell numbers in WT and *mTOR*^*f/f*^-*CD4Cre* mice 14–16 days after *C. rodentium* infection. Each circle represents one mouse. Data shown are representative or calculated from at least five experiments except Fig. 2H, which are calculated from two experiments. *P < 0.05; **P < 0.01; ***P < 0.001 determined by unpaired two-tailed Student *t*-test.

To determine whether mTOR deficiency affected T-cell populations that are important for resistance to *C. rodentium*, we assessed those cells in the LP of the small and large intestines (SI-LP and LI-LP, respectively) in both naïve mice and those infected with the bacterium. We found that the percentages and total numbers of αβT-cells were severely reduced in SI-LP and LI-LP in *mTOR*^*f/f*^-*CD4Cre* mice (Fig. 2A–C). The decrease occurred in both CD4 and CD8 αβT-cells (Fig. 2D), although the effect was greater in the CD8 αβT-cell compartment (Fig. 2E). In *mTOR*^*f/f*^-*CD4Cre* mice, there were marked decreases in CD44^+^CD62^−^ effector memory T (T_EM_) cells, whereas CD44^+^CD62^+^ central memory T (T_CM_) cells and CD44^−^CD62^+^ naïve T (T_N_) cells were relatively more rare and less affected (Fig. 2F,G). *mTOR*^*f/f*^-*CD4Cre* mice that were infected with *C. rodentium* displayed similar skewing of the mucosal T-cell populations as did naïve mice (Fig. 2H). Together, these observations revealed a crucial role of mTOR for LP T-cell accumulation in both small and large intestines under steady-state and inflammatory conditions with CD8 T-cells displaying the greater effect of mTOR deficiency.

### Roles of mTORC1 and mTORC2 in T-cell accumulation in the intestines

**Figure 3:**
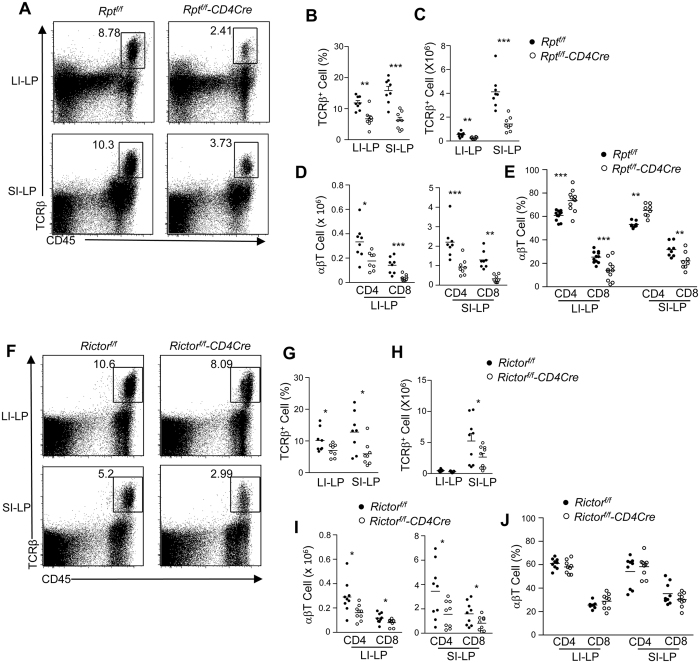
Effects of mTORC1 or mTORC2 deficiency on T-cell numbers in the LP compartments of the intestines. SI-LP and LI-LP single cell preparations from *Rptor*^*f/f*^ and *Rptor*^*f/f*^-*CD4Cre* mice (**A–E**) or *Rictor*^*f/f*^ and *Rictor*^*f/f*^-*CD4Cre* mice (**F–J**) were stained and analyzed similarly as in Fig. 2. (**A**–**E**) *Rptor*^*f/f*^ and *Rptor*^*f/f*^-*CD4Cre* mice. (**A**) Representative dot-plots shown CD45 vs TCRβ staining. (**B**,**C**) Scatter graphs showing the mean ± SEM of αβT-cell percentages within CD45^+^ cells (**B**) and numbers (**C**,**D**) CD4 and CD8 T-cell numbers. (**E**) Percentages of CD4 and CD8 subsets within αβT-cells. (**F**–**J**) *Rictor*^*f/f*^ and *Rictor*^*f/f*^-*CD4Cre* mice. Representative dot-plots shown CD45 vs TCRβ staining. (**G**,**H**) Scatter graphs showing the mean ± SEM of αβT-cell percentages within CD45^+^ cells (G) and numbers (**H**,**I**) CD4 and CD8 T-cell numbers. (**J**) Percentages of CD4 and CD8 subsets within αβT-cells. Each circle represents one mouse. Data shown are representative or calculated from at least five experiments. *P < 0.05; **P < 0.01; ***P < 0.001 determined by unpaired two-tailed Student *t*-test.

mTOR signaling is mainly mediated by mTORC1 and mTORC2^21^. To dissect the roles of mTORC1 and mTORC2 on T-cell maintenance in the gut, we examined *Rptor*^*f/f*^*-CD4Cre* (mTORC1KO; Fig. 3A–E) and *Rictor*^*f/f*^*-CD4Cre* (mTORC2KO; Fig. 3F–J) mice. LI-LP and SI-LP αβT-cells in mTORC1KO mice were decreased in both percentage (Fig. 3A,B) and number (Fig. 3C) compared with WT controls, although the reductions were less severe than those in the mTORKO mice. Similar to mTORKO mice, the reductions occurred in both CD4 and CD8 subsets in mTORC1KO mice (Fig. 3D), with CD8 T-cells being more severely affected than CD4 T-cells (Fig. 3E). In mTORC2KO mice, αβT-cell percentages were decreased in both LI-LP and SI-LP compartments (Fig. 3F,G), with total αβT-cells as well as CD4^+^ and CD8^+^ subsets being decreased by about 50% (Fig. 3H,I). However, the magnitude of decreases in Rictor/mTORC2 deficient mice was less severe than Raptor/mTORC1-deficient mice. Moreover, as opposed to the mTORKO and mTORC1KO mice, SI-LP and LI-LP CD4 and CD8 T-cell percentages within αβT-cells were not obviously skewed in Rictor/mTORC2KO mice (Fig. 3J). Together, these observations indicate that both mTORC1 and mTORC2 contributed to αβT-cell accumulation in both SI-LP and LI-LP compartments and that mTORC1 appears to play a somewhat more important role than mTORC2. Because a deficiency of either mTORC1 or mTORC2 did not fully recapitulate the severity of intestinal T-cell scarcity observed in mTORKO mice, these observations also suggest that these two complexes might synergistically promote αβT-cell accumulation in the SI-LP and LI-LP compartments.

### Reduced generation of gut-trophic T-cells in mLNs and PPs in mTOR deficient mice

**Figure 4:**
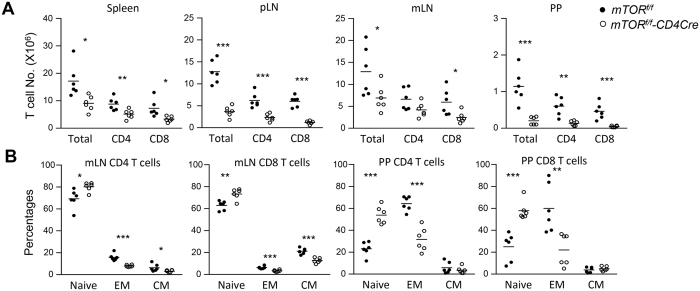
Decreased effector T-cells in mLNs and PPs in the absence of mTOR. PP and mLN single cell preparations from *mTOR*^*f/f*^ and *mTOR*^*f/f*^-*CD4Cre* mice were stained for CD4, CD8, CD44, CD62L and TCRβ followed by flow-cytometric analysis. (**A**) Scatter graphs showing the mean ± SEM of total T, CD4, and CD8 T-cell numbers in the spleen, pLNs, mLNs, and PPs from *mTOR*^*f/f*^*-CD4Cre* and control mice. (**B**) Percentages of CD4 and CD8 CD44^−^CD62L^+^ naïve, CD44^+^CD62L^−^ effector memory (EM), and CD44^+^CD62L^+^ central memory (CM) T-cells in mLNs and PPs from *mTOR*^*f/f*^*-CD4Cre* and control mice. Each circle represents one mouse. Data shown are calculated from at least five experiments. *P < 0.05; **P < 0.01; ***P < 0.001 determined by unpaired two-tailed Student *t*-test.

T-cells acquire gut-homing properties in the gut-associated lymphoid organs such as PPs and mLNs. In these organs, naïve T-cells are activated by antigens derived from commensal microbes to become CD44^+^ effector cells. These cells downregulate CCR7 but upregulate CCR9, CD103, and ItgαEβ7, which are important for T-cell homing to the intestines^5^,^6^,^7^,^8^. As shown in Fig. 4A, total T-cells as well as CD4 and CD8 T-cell subsets in the spleen, peripheral lymph nodes (pLNs), mLNs, and PPs were decreased in *mTOR*^*f/f*^*-CD4Cre* mice compared with control mice. Moreover, within CD4 and CD8 T-cells, the relative ratios of CD44^−^CD62L^+^ naïve T-cells were increased, but the CD44^+^CD62L^−^ effector memory (EM) T-cells were decreased in mLNs and PPs of mTOR-deficient mice (Fig. 4B). CD44^+^CD62L^+^ central memory (CM) CD4 and CD8 T-cell percentages were decreased in mLNs but not in PPs in mTOR-deficient mice. Although decreased in numbers, mTOR-deficient EM and CM CD4 and CD8 T-cells expressed similar levels of CCR9, CD103, and integrin α4β7 (Supplementary Fig. S1) compared with their controls. Together, these observations suggested that mTOR deficiency reduced generation of gut-homing T-cells in PPs and mLNs without obviously affecting expression of gut-homing molecules on these cells.

### mTORC1 but not mTORC2 deficiency impaired SI-LP and LI-LP T-cell homeostatic proliferation

**Figure 5:**
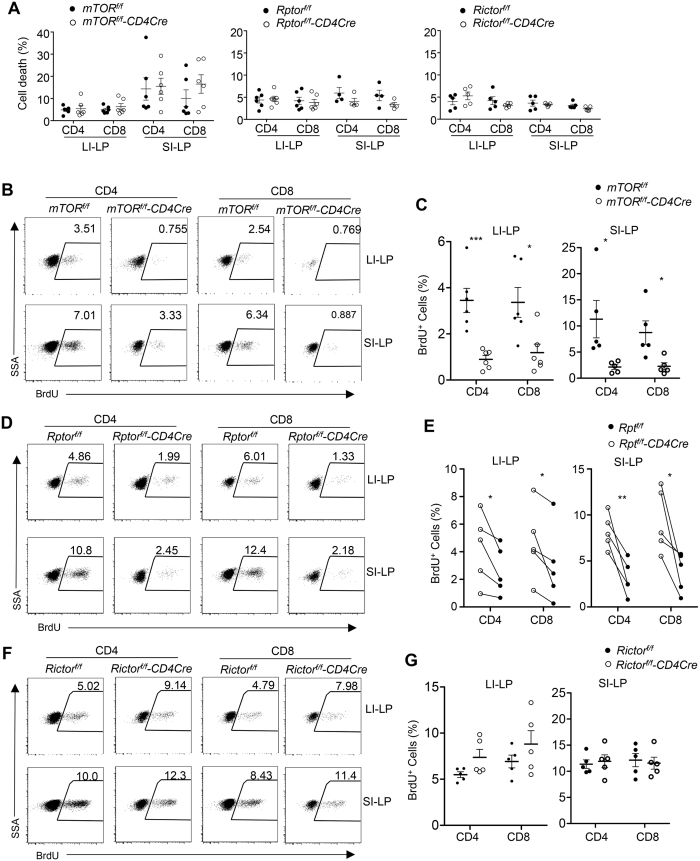
Differential role of mTORC1 and mTORC2 in proliferative renewal of SI-LP and LI-LP T-cells. (**A**) Death rates of SI-LP and LI-LP T-cells in *mTOR*^*f/f*^*-CD4Cre, Rptor*^*f/f*^*-CD4Cre, Rictor*^*f/f*^*-CD4Cre,* and their corresponding control mice determined by staining with a LIVE/DEAD® Fixable Dead Cell Stain. (**B**–**G**) BrdU incorporation in SI-LP and LI-LP T-cells. *mTOR*^*f/f*^*-CD4Cre* and *mTOR*^*f/f*^ mice (**B**,**C**), *Rptor*^*f/f*^*-CD4Cre* and *Rptor*^*f/f*^ mice (**D**,**E**), and *Rictor*^*f/f*^*-CD4Cre* and *Rictor*^*f/f*^ mice (**F**,**G**) were intraperitoneally (*i.p*) injected with BrdU and BrdU incorporation in SI-LP and LI-LP T-cells were examined by flow-cytometry 16 hours later. (**B**,**D**,**F**) Representative dot-plots showing BrdU staining in gated SI-LP and LI-LP CD4 and CD8 T-cells. (**C**,**E**,**G**) Scatter plots showing percentages of BrdU^+^ T-cells in the indicated populations. Horizontal bars represent the mean ± SEM. Each circle represents one mouse of the indicated genotypes. Data shown represent or calculated from at least three experiments. *P < 0.05; **P < 0.01; ***P < 0.001 determined by paired (E) and unpaired (others) two-tailed Student *t*-test.

T-cell survival in the local environment and *in situ* proliferation could influence their homeostasis in the intestines. mTOR, mTORC1, or mTORC2 deficiency did not obviously affect SI-LP and LI-LP CD4 and CD8 T-cell survival (Fig. 5A). However, both LI-LP and SI-LP T-cells from *mTOR*^*f/f*^*-CD4Cre* mice incorporated much less BrdU than controls in the steady state (Fig. 5B,C), suggesting impaired proliferative renewal of SI-LP and LI-LP T-cells in the absence of mTOR. Further analysis revealed that *Rptor*^*f/f*^*-CD4Cre* SI-LP and LI-LP T-cells (Fig. 5D,E) but not *Rictor*^*f/f*^*-CD4Cre* SI-LP and LI-LP T-cells (Fig. 5F,G) incorporated less BrdU than their controls. Together, these observations revealed that a significant portion of αβT-cells in both the LI-LP and SI-LP compartments proliferated in the steady state in an mTOR-dependent manner to maintain their homeostasis, and mTORC1 but not mTORC2 was critical for such proliferative renewal.

### mTORC1 is required for T-cell homeostasis after establishing intestinal residency

**Figure 6:**
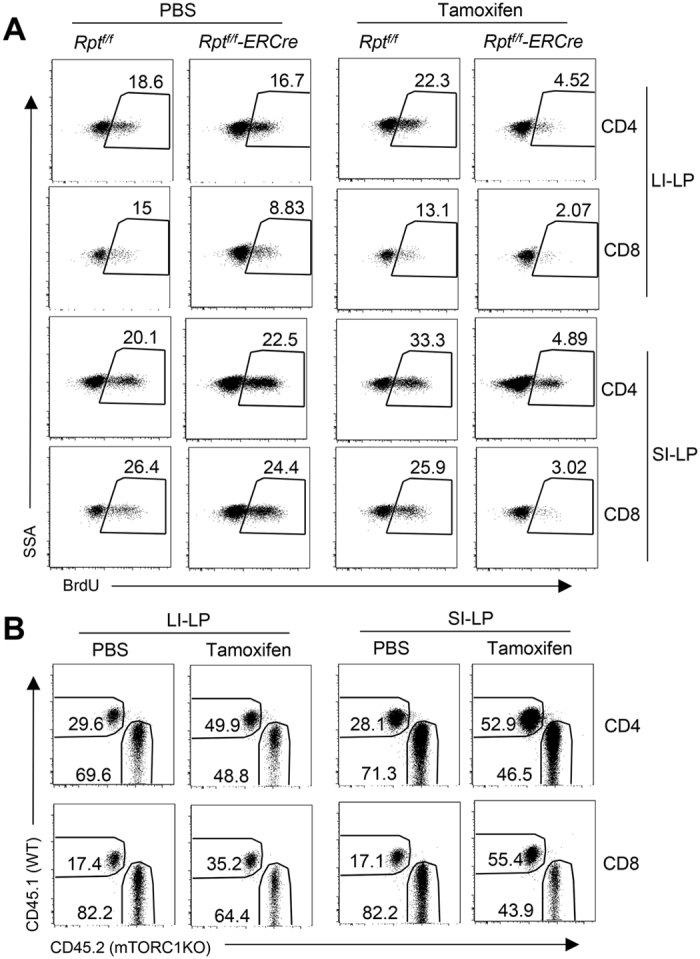
Assessment of mTORC1 in SI-LP and LI-LP T-cell proliferative renewal after acute mTORC1 ablation in mature T-cells. T-cells isolated from CD45.2^+^*Rptor*^*f/f*^*-CreER* and CD45.1^+^ WT mice were intravenously injected as a mixture into *Rag2*^−/−^ mice. One month later, recipient mice were *i.p.* injected with tamoxifen or PBS on days 1, 2, 5, and 12, and with BrdU on day 14. Mice were euthanized on day 15 for assessment of BrdU incorporation. Shown are: (**A**) Representative dot-plots of BrdU staining in SI-LP and LI-LP CD4 and CD8 T-cell subsets in gated CD45.1^+^TCRβ^+^ WT and CD45.2^+^TCRβ^+^ *Rptor*^*f/f*^*-CreER* T-cells. (**B**) CD45.1 and CD45.2 expression in gated TCRβ^+^ SI-LP and LI-LP cells. Data shown are representative of two experiments.

Because mTORC1 deletion began in thymocytes in *Rptor*^*f/f*^*-CD4Cre* mice, decreased SI-LP and LI-LP T-cell BrdU incorporation in these mice could be caused by an altered local environment or properties that imprinted on T-cells before they resided in the gut. To examine if mTORC1 is important for T-cell homeostatic renewal after establishing residency in SI-LP and LI-LP, we adoptively transferred a mixture of CD45.2^+^*Rptor*^*f/f*^*-CreER* T-cells and CD45.1^+^ WT T-cells into Rag2-deficient mice. One month after transfer, recipient mice were administrated with either tamoxifen or PBS on days 1, 2, 5, and 12 to delete *Rptor*; injected with BrdU on day 14; and euthanized for examination on day 15. Similar to *Rptor*^*f/f*^*-CD4Cre* mice, BrdU incorporation in SI-LP and LI-LP CD4 and CD8 T-cells in *Rptor*^*f/f*^*-CreER* mice were obviously decreased in tamoxifen but not PBS-injected mice (Fig. 6A). Correlated with decreased homeostatic proliferation, *Rptor*^*f/f*^*-CreER* T-cell to WT T-cell ratios were decreased in tamoxifen-injected recipients compared with PBS-injected chimeric mice (Fig. 6B). These observations further support that mTORC1 promotes SI-LP and LI-LP CD4 and CD8 T-cell proliferative renewal to maintain T-cell homeostasis in the gut.

## Discussion

T-cells that reside in mucosal tissues play a number of important roles in host defense against microbial infections such as *C. rodentium* and *E. coli*^1^,^2^,^47^. Using mice with T-cell-specific deficiency of mTOR, we provide clear evidence that mTOR is crucial for both CD4 and CD8 T-cell accumulation in the SI-LP and LI-LP compartments and for promoting host defense against the bacterial pathogen *C. rodentium*. We demonstrated that mTOR-deficient mice exhibit severe decreases in both SI-LP and LI-LP CD4 and CD8 T-cell numbers under steady-state conditions. We have also shown that both CD4 and CD8 T-cell numbers in the intestines are greatly reduced in mTOR-deficient mice as compared to control mice following *C. rodentium* infection. These observations suggest that mTOR plays an important role in generating resident T-cells within the intestinal mucosa under inflammatory conditions, as a means to mount an effective immune response. A recent report found that decreased mTORC1 activities reduce the presence of resident CD8 T-cells in the SI-LP following viral infection^46^. We have found that a T-cell-specific deficiency in either Raptor/mTORC1 or in Rictor/mTORC2 leads to decreases in both CD4 and CD8 T-cells in the LP compartments in both small and large intestines. Our data provide genetic evidence that not only supports but also extends the role of both mTORC1 and mTORC2 for T-cell accumulation in both the SI-LP and LI-LP compartments under steady-state conditions.

An important question is how mTOR controls T-cell numbers in the intestines. A previous report has suggested that the PI3K-mTOR axis controls T-cell trafficking via regulating KLF2, CD62L, and CCR7^48^. Upregulation of CCR9, CD103, and integrin α_4_β_7_ in activated T-cells in PPs and mLNs is an important step that imprints T-cells for homing to the intestines^9^,^12^,^13^,^14^,^15^. However, we did not observe overt differences in the expression of these homing molecules in mTOR-deficient CD4 and CD8 T-cells, suggesting that mTOR may not directly regulate expression of these molecules in T-cells. At present, we cannot rule out that mTOR may participate in relaying signals from these gut-homing molecules and it may also play an important role in promoting the retention of resident T-cells within the intestines.

Our data suggest that one of the reasons for decreased T-cell accumulation in mTOR-deficient mice is the decreased generation of effector T-cells within PPs and mLNs. In *mTOR*^*f/f*^*-CD4Cre* mice, both CD4 and CD8 T-cell numbers are overtly decreased in mLNs and PPs, along with decreased CD62L^−^CD44^+^ effector T-cell percentages. Thus, although mTOR-deficient effector T-cells express normal levels of gut-homing molecules, their dramatically decreased numbers may directly lead to decreased T-cell migration to the intestines. Another reason for decreased T-cell numbers in the intestines in the absence of mTOR may be decreased proliferative renewal of T-cells that already reside within the gut. In the current study, we have demonstrated that mTOR- or Rptor/mTORC1-deficient T-cells but not Rictor/mTORC2-deficient SI-LP and LI-LP T-cells incorporate less BrdU compared with their WT controls. These results suggest that mTORC1 but not mTORC2 plays a crucial role in T-cell expansion within the local environment. Using CreER transgenic mice and tamoxifen treatment, we also found that ablation of mTORC1 within T-cells that already take residency in intestinal LP compartments results in decreases in both CD4 and CD8 T-cells in these locations. Because these decreases are correlated with decreased proliferation, it suggests that T-cells in the SI-LP and LI-LP compartments undergo constant proliferative renewal in an mTORC1-dependent manner. This requirement of mTORC1 suggests that mTOR is likely activated in these cells in the steady state. Further studies should determine how mTOR is activated under these conditions and regulated in resident SI-LP and LI-LP T-cells under both steady state and in inflammatory conditions.

A previous study found that CD3^+^ T cells were only slightly decreased in the spleen but not LNs in *mTOR*^*f/f*^*-CD4Cre* mice^49^. Our data demonstrated that both CD4 and CD8 T-cell numbers were decreased not only in PPs and mLNs but also in the spleen and pLNs in *mTOR*^*f/f*^*-CD4Cre* mice. At present, the reason for discrepancies of peripheral T-cell numbers in mTOR- and mTORC1/2-deficient mice among different studies is not clear. It is possible that differences in local environment might contribute to differences in revealing the effects of mTOR in peripheral T-cell homeostasis. Correlated with decreased T-cell numbers, we also found that T-cells in *mTOR*^*f/f*^*-CD4Cre* mice incorporate less BrdU than their WT counterparts in the steady state (Supplementary Fig. S2). Our data revealed that mTOR was involved in T-cell homeostatic proliferation in peripheral lymphoid organs and that impaired homeostatic proliferation of mTOR-deficient T-cells might contribute to their decreases in these organs. Although Raptor/mTORC1 deficiency only caused decreases of CD8 T-cells in pLNs, mLNs, and PPs in *Rptor*^*f/f*^*-CD4Cre* mice (Supplementary Fig. S3), which was consistent with a previous report^36^, in irradiation chimeric mice reconstituted with CD45.1^+^ WT and CD45.2^+^ *Rptor*^*f/f*^*-CD4Cre* bone marrow cells at a 1:1 ratio, *Rptor*^*f/f*^*-CD4Cre*-derived T cells, but not B220^+^ B cells or B220^+^CD4^−^CD8^−^ non-T/B cells, were underrepresented in the spleen compared with WT-derived counterparts, revealing a previously unappreciated role of mTORC1 for CD4 T-cell homeostasis. Rictor/mTORC2 deficiency in *Rictor*^*f/f*^*-CD4Cre* mice led to decreases of both CD4 and CD8 T-cells in peripheral lymphoid organs (Supplementary Fig. S4), which was consistent previous reports demonstrating an important role of Rictor/mTORC2 for T cell developments and^50^,^51^,^52^. However, such decreases in mTORC1 or mTORC2 deficient mice were not as severe as in mTOR-deficient mice, suggesting that mTORC1 and mTORC2 play a synergistic role in promoting T-cell homeostasis in peripheral lymphoid organs.

mTOR inhibition has been shown to be beneficial under many pathological and physiological conditions. mTOR inhibitors have been utilized extensively for cancer patients and for inducing tolerance after transplantation. A caloric restriction inhibits mTOR, and the inhibition of mTOR prolongs life span^53^. mTOR inhibition enhances memory CD8 T-cell responses to pathogens and vaccines (21). The finding that mTOR is also important for T-cell accumulation in the intestines suggests that mTOR inhibition can also be a potential therapeutic strategy to treat intestinal diseases, such as inflammatory bowel diseases, in which T-cells play an important role. While the benefits of mTOR inhibition can be tremendous, potential side effects of mTOR inhibition on the immune system such as thymic atrophy^25^,^26^, impaired T-cell and iNKT-cell function, and decreased mucosal immunity (ref. 46 and this study) should also be considered.

## Methods

### Mice

*mTOR*^*f/f*^*^-^*54, *Rptor*^*f/*^^*f*-^55, *Rictor*^*f/*^^*f*^-56, and *Rag-2*-deficient mice were purchased from the Jackson laboratory. The *mTOR*^*f/f*^*, Rptor*^*f/f*^, and *Rictor*^*f/f*^ mice had been further backcrossed to C57Bl/6J background for at least four generations after acquisition. *CD4Cre* mice^57^ were purchased from Taconic Inc. Rosa26-CreER mice were previously reported^58^. All animals were housed in specific pathogen-free conditions. This study was carried out in strict accordance with the recommendations in the Guide for the Care and Use of Laboratory Animals of the National Institutes of Health. The experiments described were approved by the Institutional Animal Care and Use Committee of Duke University.

### Reagents and antibodies

Iscove’s modified Dulbecco’s medium (IMDM) was supplemented with 10% (vol/vol) FBS, penicillin/streptomycin, and 50 μM 2-mercaptoethanol (IMDM-10). Cell death was determined by adding the Live/Dead Fixable Violet Dead Cell Stain (Invitrogen) according to the manufacturer’s protocol and was analyzed by flow cytometry. Fluorescence-conjugated anti-mouse CD4 (GK1.5), CD8α (53–6.7), CD8β (53–5.8), TCRβ (H57-597), CD3 (145-2c11), CD25 (PC61), CD44 (IM7), CD62L (MEL-14), CD45 (30-F11), CD45.1 (A20), CD45.2 (104), ITGb7 (FIB27), CD103 (2E7), and CCR9 (9B1) antibodies were purchased from BioLegend.

### SI-LP and LI-LP single cell preparation

SI-LP and LI-LP single cell suspension for large and small intestines were prepared as previously reported with modification^59^,^60^. Briefly, small and large intestines were flushed and washed with PBS. Fat tissues and PPs were removed from the intestines. Both the small intestine and colon were cut longitudinally and laterally into 0.5 cm pieces, which were placed in 20 ml (small intestine) and 5 ml (colon) pre-warmed IELs buffer (PBS with 10% FBS, 5 mM EDTA, and 1 mM DTT) and incubated at 37 °C for 30 minutes in a shaking incubator (200 rpm). Samples were then vortexed vigorously for 15 seconds and filtered through cell strainers. Tissues remained in the strainers were incubated in the IEL buffer for another 30 minutes and re-filtered. Strained tissues were further cut into small pieces that were placed in 10 ml (small intestine) or 5 ml (colon) pre-warmed digestion buffer (IMDM with 10% FBS, 1.5 mg/ml collagenase IV, and 0.5 mg/ml DNase I) and incubated at 37 °C, 200 rpm for 30 minutes. After digestion, samples were filtered through cell strainers to collect SI-LP and LI-LP cells. SI-LP and LI-LP preparations were centrifuged and washed with IMDM twice and re-suspended in IMDM-10.

### Flow Cytometry

All flow cytometry data were collected using a FACS Canto-II (BD Biosciences) and analyzed with FlowJo software.

### BrdU incorporation

For *in vivo* cell proliferation analysis, mice were intraperitoneally (i.p.) injected with 1.5 mg of BrdU in 100 μl PBS. Sixteen hours later, single-cell suspensions were made, and cells were stained for cell surface expression of CD4, CD8, and TCRβ. Afterward, samples were intracellularly stained for BrdU using an FITC BrdU Flow Kit (BD Pharmingen) as previously described^25^.

### *C. rodentium* infection

Mice were infected with *C. rodentium* as previously described^3^. Briefly, the *C. rodentium* was grown in LB medium containing streptomycin overnight at 37 °C with shaking. Mice were intragastrically injected with 7 × 10^7^ CFU *C. rodentium* in 0.2 ml PBS. Mice were weighed daily after infection to monitor the disease development and euthanized 14–16 days after infection.

### Statistical analysis

Data are presented as mean ± SEM, and statistical significance was determined by two-tailed Student’s *t* test. Kaplan-Meyer survival analysis was determined by the Log-rank (Mantel-Cox) test. The P values are defined as follows: *p < 0.05, **p < 0.01, ***p < 0.001.

## Additional Information

**How to cite this article**: Lin, X. *et al.* mTOR is critical for intestinal T-cell homeostasis and resistance to *Citrobacter rodentium. Sci. Rep.* **6**, 34939; doi: 10.1038/srep34939 (2016).

## Supplementary Material

Supplementary Information
