# Supplementary material for: mTOR is critical for intestinal T-cell homeostasis and resistance to Citrobacter rodentium
Source: Sci Rep. 2016 Oct 12;6:34939. doi: 10.1038/srep34939 (PMC5059740; doi:10.1038/srep34939)
Supplement: Supplementary Information [file srep34939-s1.pdf]

Supplementary Fig. S1

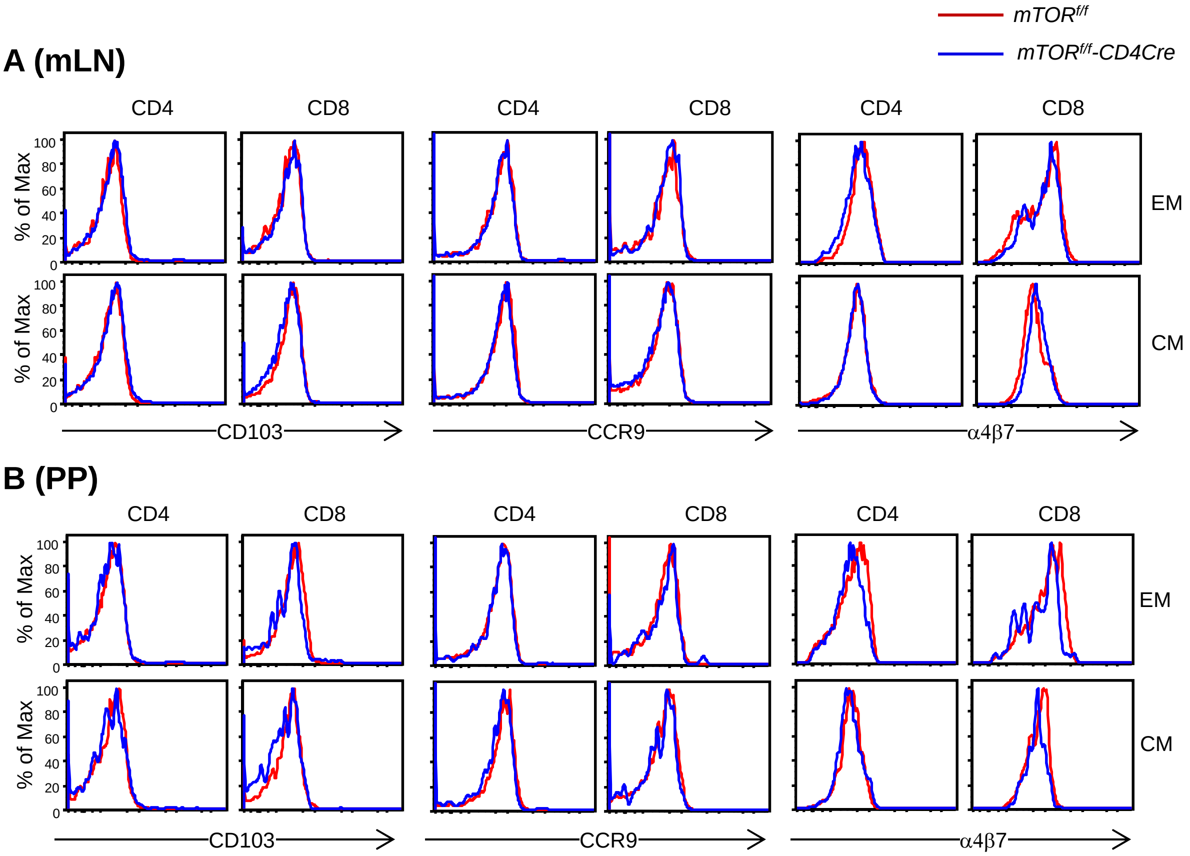

Figure S1. Expression of gut-trophic molecules on effector CD4 and CD8 T cells in the mLNs and PPs in *mTOR$^{f/f}$-CD4Cre* and mTOR$^{f/f}$ mice. Overlaid histograms show CD103, CCR9, and integrin α4β7 staining in gated CD44^{+}CD62L^{+} (CM) and CD44^{+}CD62L^{-} (EM) CD4 and CD8 T cells. Data shown are representative of three experiments.

Supplementary Fig. S2

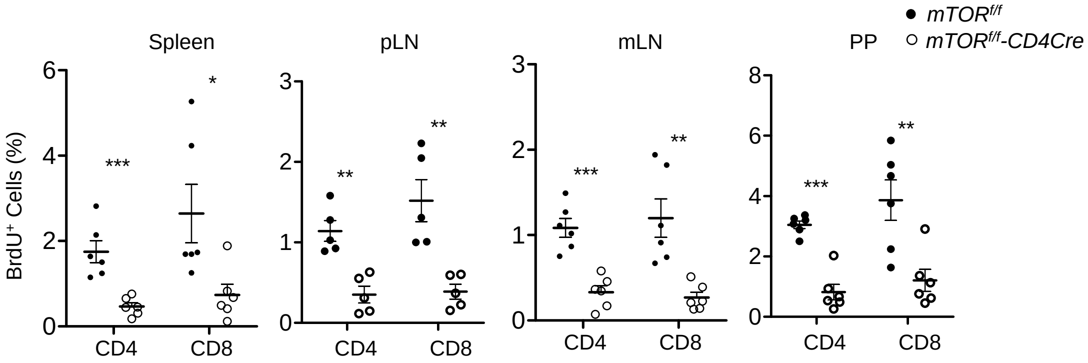

Figure S2. BrdU incorporation in CD4 and CD8 T cells in the peripheral lymphoid organs from *mTOR$^{f/f}$-CD4Cre* and *mTOR$^{f/f}$* mice. Mice were intraperitoneally (*i.p*) injected with BrdU and BrdU incorporation in T-cells were examined by flow-cytometry 16 hours later. Scatter plots showing percentages of BrdU^{+} T-cells in the indicated populations. Horizontal bars represent the mean ± SEM. Each circle represents one mouse of the indicated genotypes. Data shown represent or calculated from at least three experiments. *, P<0.05; **, P<0.01; ***, P<0.001 determined by unpaired two-tailed Student t-test.

Supplementary Fig. S3

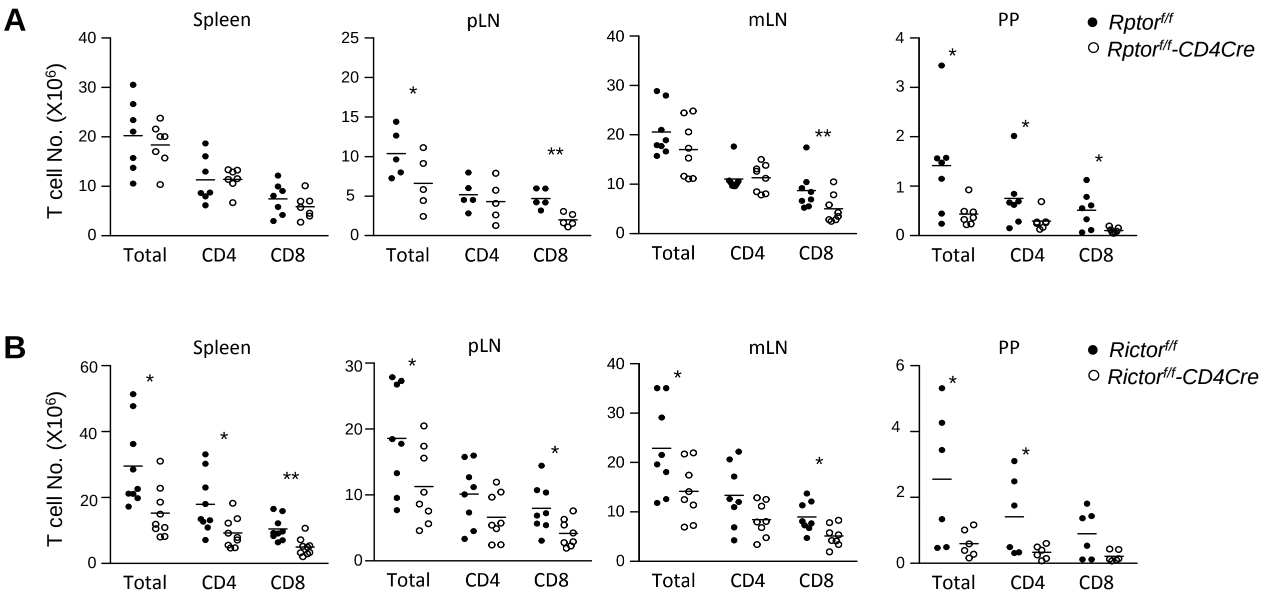

Figure S3. Effects of mTORC1 and mTORC2 defciency on T-cell numbers in peripheral lymphoid organs. Scatter graphs showing the mean ± SEM of total T, CD4, and CD8 T-cell numbers in the spleen, pLNs, mLNs, and PPs from *Raptor$^{f/f}$* and *Raptor$^{f/f}$-CD4Cre* mice (**A**) or *Rictor$^{f/f}$* and *Rictor$^{f/f}$-CD4Cre* mice (**B**) mice. Data shown are calculated from at least five experiments. *, P<0.05; **, P<0.01 determined by unpaired two-tailed Student t-test.

Supplementary Fig. S4

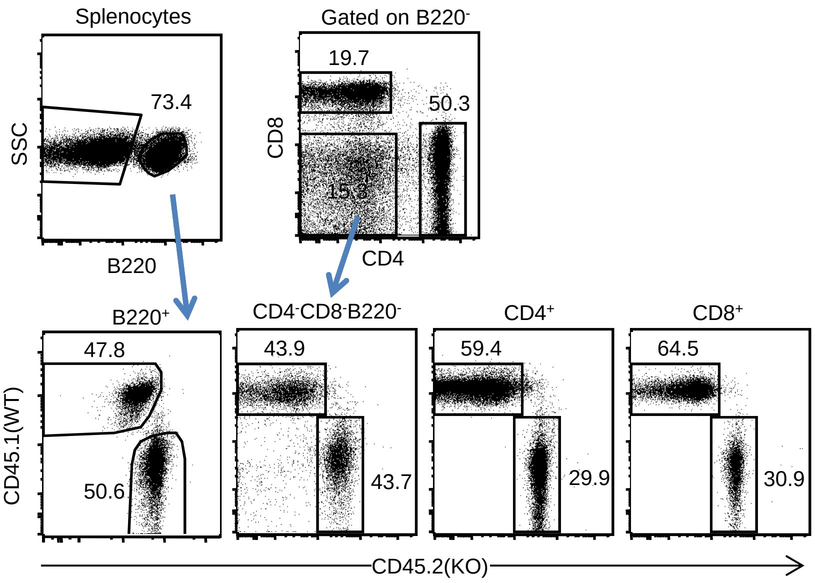

Figure S4. Role of mTORC1 in splenic T cell homeostasis in mixed bone marrow irradiation chimeric mice. WT (CD45.1^{+}) and *Raptor$^{f/f}$-CD4Cre* (CD45.2^{+}) BM cells were mixed at a 1:1 ratio and adoptively transferred into sublethally irradiated *TCR*-$\beta^{-/-}\delta^{-/-}$ mice. Eight weeks later, splenocytes from the chimeric mice were analyzed for B220^{+} B cells, CD4 and CD8 T-cell and B220^{-}CD4^{-}CD8^{-} non-T/B cells. Data shown are representative of three experiments.
